# 

**DOI:** 10.1192/bjb.2017.20

**Published:** 2018-08

**Authors:** Jennie Parker

**Affiliations:** Service User Researcher and Knowledge and Understanding Framework (KUF) Trainer, London, UK; email: jennie.parker@btinternet.com

According to Dr Lee personality disorder is ‘not your fault’ and is a treatable ‘disorder’ for individuals that are both able and willing to become involved with available therapeutic programmes. This book immediately highlights the importance of responsibility, not only on the part of clinical staff, but also for the person engaging with services, and as someone who has lived with the diagnosis of borderline personality disorder for many years, I was pleased to find that a key theme was one of empowerment.

The format and content allows a wide reach in terms of audience, being accessible to both clinicians and those with lived experience, their families and others with whom they may come into contact. The author utilises well the fictional character Nina to portray real-life examples, while not apportioning blame, nor attributing her difficulties to one particular aspect of her life. Although the focus is mainly on borderline personality disorder, the use of case examples in a chapter dedicated to other personality disorders gives an excellent insight into how these may present. Indeed, within this, is also the notion of the enduring and pervasive nature of personality disorder, how it affects lives, and the ability to question whether a label is indeed always helpful or necessary.

Lee uses the term ‘personality configurations’ to describe individual differences and how carers may adapt to a role which enables the individual. The label of personality disorder is often perceived as stigmatising and, therefore, reframing how this is presented to others can be significant in accepting both the diagnosis and how to manage it. Overall, he attempts to reduce stigma and the feeling of being stigmatised, while retaining an honest approach to how negative perceptions can influence relationships of all levels. This notion of relationships is also addressed, being key to personality disorder and to therapeutic alliance.

The information on the differences between borderline personality disorder and other psychiatric diagnoses may help to dispel some myths. For example, Lee suggests that clinical depression and depressive symptoms of borderline personality disorder are not the same. Although this comes across as a medicalised view, it fits well with my personal experience where unnecessary pharmacological treatment pathways were the only option given, rather than an effective therapeutic management of negative emotion.

The chapter on treatments provides a comprehensive overview of evidence-based therapies for borderline personality disorder and indirectly highlights the paucity of evidence-based options for other personality disorder classifications. From the range of treatment options available, albeit limited, the information given is easy to digest, providing key concepts and a tabulated summary of areas such as goals and the all-important patient-therapist relationship.

At one point Lee outlines the role of choice in finding a treatment programme and therapist. In an ideal world, the choice of therapist can be an essential aspect of engagement in services but in reality this is not always the case – this is perhaps an area towards which health care services can move their focus.

I would have welcomed discussion around the role of gender in personality disorder, in relation to both diagnosis and treatment outcomes, as well as a more explicit and detailed examination around the role of emotion (and how this underlies relationships and day-to-day or even minute-to-minute interactions). Although this idea is present in the examples used, further elaboration may be helpful.

For me, the book summarises a positive approach to understanding and helping individuals with personality disorder without leaving the reader feeling helpless; this is achieved not by changing who we are but, as the author suggests, by building on the positive qualities we already have. I liked the idea that the final chapters provide an ending, not just for Nina but for the reader.

Taking a collaborative approach to treatment goals and enabling individuals to have a sense of agency in their passage to self-discovery is an essential aspect of the book – and a way of working that I found to be imperative to discovering a way through what for me was a confusing, frustrating and distressing journey.

